# Judicial Opinions 133 and 134

**DOI:** 10.1099/ijsem.0.007203

**Published:** 2026-06-24

**Authors:** David R. Arahal, Carolee T. Bull, Henrik Christensen, Maria Chuvochina, Christopher Dunlap, Markus Göker, Maria del Carmen Montero Calasanz, Valerie A. Skye, Peter Vandamme, Antonio Ventosa, Stefano Ventura, Peter Young

**Affiliations:** 1 Departamento de Microbiología y Ecología, Universitat de València, Valencia, Spain; 2 Department of Plant Pathology and Environmental Microbiology, Pennsylvania State University, 211 Buckhout Lab, University Park, PA 16802, USA; 3 Department of Veterinary and Animal Sciences, University of Copenhagen, Stigbøjlen 4, 1870 Frederiksberg C, Denmark; 4 Center for Microbial Communities, Department of Chemistry and Bioscience, Aalborg University, Fredrik Bajers Vej 7H, 9220 Aalborg East, Denmark; 5 Crop Bioprotection Research Unit, USDA/ARS/NCAUR, 1815 N. University St, 61604 Peoria, Illinois, USA; 6 Leibniz Institute DSMZ – German Collection of Microorganisms and Cell Cultures, Inhoffenstrasse 7B, D-38124 Braunschweig, Germany; 7 IFAPA Las Torres - Andalusian Institute of Agricultural and Fisheries Research and Training, Cra. Sevilla-Cazalla de la Sierra, 41200, Alcalá del Río, Sevilla, Spain; 8 Lawrence Berkeley National Laboratory - Joint Genome Institute, Berkeley, CA 94720, USA; 9 Laboratorium voor Microbiologie, Universiteit Gent, K. L. Ledeganckstraat 35, 9000 Gent, Belgium; 10 Department of Microbiology and Parasitology, Faculty of Pharmacy, University of Sevilla, Sevilla, C/. Prof. Garcia Gonzalez 2, ES-41012 Sevilla, Spain; 11 IRET-CNR, Research Institute on Terrestrial Ecosystems, National Research Council, Via Madonna del Piano 10, 50019 Sesto Fiorentino, and NBFC, National Biodiversity Future Center, Palermo, Italy; 12 Department of Biology, University of York, York YO10 5DD, UK

**Keywords:** acidophilic bacteria, Approved Lists, Candidatus, enteric group, human pathogens, methane-oxidizing bacteria, Providence group, taxonomy

## Abstract

Opinion 133 addresses the unusual situation of two genus names that are homonyms and are also considered heterotypic synonyms. Furthermore, it clarifies the meaning of the term ‘homonym’ in the International Code of Nomenclature of Prokaryotes, as well as the implications of the absence of the term ‘isonym’ from that code. Isonyms are homotypic synonyms with the same spelling and as such are adequately regulated by the code. The Judicial Commission agrees that the names *Methylacidiphilum caldifontis* Awala *et al*. 2023, *Methylacidiphilum* Awala *et al*. 2023, *Methylacidiphilaceae* Awala *et al*. 2023 and *Methylacidiphilales* Awala *et al*. 2023 are illegitimate. The possible actions to resolve this illegitimacy are outlined. The request to conserve *Methylacidiphilum* Awala *et al*. 2023 over *Methylacidiphilum* Ratnadevi *et al*. 2023 is not granted. However, *Methylacidiphilaceae* Awala *et al*. 2023 and *Methylacidiphilales* Awala *et al*. 2023 are conserved with *Methylacidiphilum* Ratnadevi *et al*. 2023 as the type genus, thereby rendering them legitimate. Opinion 134 addresses two distinct enquiries arising from the same Request for an Opinion. The Judicial Commission confirms that the priority between *Yokenella regensburgei* Kosako *et al*. 1985 and its heterotypic synonym *Koserella trabulsii* Hickman-Brenner *et al*. 1985 may at present be unresolved. Considering previous statements by the Judicial Commission, the differences in the amount of usage between the two names and their medical relevance, the request is granted. The name *Yokenella* Kosako *et al*. 1985 is conserved over *Koserella* Hickman-Brenner *et al*. 1985, as is the epithet in *Yokenella regensburgei* Kosako *et al*. 1985 over the epithet in *Koserella trabulsii* Hickman-Brenner *et al*. 1985. The Judicial Commission also confirms that the epithet in *Proteus inconstans* (Ornstein 1920) Shaw and Clarke 1955 (Approved Lists 1980) has priority over the one in its homotypic synonym *Providencia alcalifaciens* (de Salles Gomes 1944) Ewing 1962 (Approved Lists 1980). However, it is doubtful whether this renders *Providencia* Ewing 1962 (Approved Lists 1980) illegitimate. There are other reasons to conserve the epithet in *Providencia alcalifaciens* (de Salles Gomes 1944) Ewing 1962 (Approved Lists 1980) over that in *Proteus inconstans* (Ornstein 1920) Shaw and Clarke 1955 (Approved Lists 1980), and this request is also granted.

## Introduction

In its guidelines for interpreting the International Code of Nomenclature of Prokaryotes (ICNP) published in 2023 [1], the Judicial Commission proposed various additions to the code to reduce the likelihood of misinterpretation:

- ‘Redundancy over inference. Some conclusions can only be inferred by combining distinct sections of the ICNP. By introducing some slight redundancy the need for complicated inferences could be reduced’.
- ‘More cross-references. Some misunderstandings can be prevented by introducing cross-references that connect to other Rules which may easily be overlooked but are relevant for the respective topic’.
- ‘More caveats. If it is already known that a certain Rule or a part thereof is sometimes misinterpreted, it makes sense to add a Note that indicates what is not meant directly to that clause’’

The main purpose of the 2025 revision of the ICNP was to compile the previous decisions of the International Committee on Systematics of Prokaryotes (ICSP) into a single document again [2]. However, many additional proposals were also made in line with the Judicial Commission’s recommendations [3]. Alongside numerous examples proposed during the public debate period [2,3], the revised code is intended to facilitate understanding for all readers, aid in its application and simplify explanations of the Judicial Commission’s decisions. Therefore, it seemed sensible to delay work on Judicial Opinion 133 slightly, in response to a Request for an Opinion by Awala *et al*. [4], to allow time to incorporate insights from the 2025 revision after its ratification [2,3]. However, none of the ratified changes are expected to fundamentally alter the content of the ICNP [5]. Judicial Opinion 134 was then added in response to a Request for an Opinion by Li [6]. Opinion 134 also benefited from the additional clarity found in the 2025 revision of the ICNP.

## Opinion 133

### The cases presented

Several years after the initial descriptions of thermoacidophilic methanotrophic bacteria belonging to the *Verrucomicrobiota* phylum [7,9], the genus *Methylacidiphilum* Ratnadevi *et al*. 2023 and its type species, *M. kamchatkense* Ratnadevi *et al*. 2023, were formally described and their names were validly published [10,11]. Soon afterwards, the names of the genus *Methylacidiphilum* Awala *et al*. 2023 and its type species, *M. caldifontis* Awala *et al*. 2023, were validly published [12,13]. However, since this publication came days after that of Methylacidiphilum by *Methylacidiphilum* by Ratnadevi *et al*. (2023) [10,11], the name Methylacidiphilum by *Methylacidiphilum* by Awala *et al*. (2023) [12,13] is a later homonym of the previous genus name. In the same publication [12,13], Awala and colleagues also described the new family *Methylacidiphilaceae* and the new order *Methylacidiphilales*, and validly published both their names.

In their Request for an Opinion, Awala *et al*. [4] ask the Judicial Commission to conserve the generic name *Methylacidiphilum* Awala *et al*. 2023 over *Methylacidiphilum* Ratnadevi *et al*. 2023, in order to resolve the presumed illegitimacy of *Methylacidiphilum* Awala *et al*. 2023 and *M. caldifontis* Awala *et al*. 2023. The authors believe that this action would also resolve the presumed illegitimacy of the names *Methylacidiphilaceae* Awala *et al*. 2023 and *Methylacidiphilales* Awala *et al*. 2023. Awala and colleagues [4] also request that the Judicial Commission confirm the accuracy of their interpretation of the terms ‘isonym’ and ‘homonym’ in relation to the names *Methylacidiphilum* Awala *et al*. 2023 and *Methylacidiphilum* Ratnadevi *et al*. 2023, as well as to names that may be generated in response to the request.

As they are a prerequisite for the subsequent arguments, the Judicial Commission will first analyse the terms ‘homonym’ and ‘isonym’ with respect to the 2025 revision of the ICNP [3]. Next, it will examine the presumed illegitimacy of the names *Methylacidiphilaceae* Awala *et al*. 2023 and *Methylacidiphilales* Awala *et al*. 2023 [12,13]. Finally, potential alternative actions to be taken if the names are found to be illegitimate will be discussed.

### The relationships between homonyms, synonyms and isonyms

Until recently, homonyms had not been explicitly defined in the ICNP [5], but they are defined in Rule 24 a of the 2025 revision [2,3] as follows:

‘*Homonyms* are names published for taxa in the same category that are spelled exactly the same (apart from orthographic or grammatical corrections, as detailed in Section 9), but which are based on different types’.

Rule 24a of the 2025 revision [2,3] defines the two kinds of synonyms envisaged by the ICNP as follows, largely in line with previous revisions [5]:

‘Synonyms may be *homotypic synonyms* (i.e. more than one name has been associated with the same type) or *heterotypic synonyms* (i.e. different names have been associated with different types that, in the opinion of the microbiologist concerned, belong to the same taxon)’.

The question arises as to whether there is a term in the ICNP that covers names that are ‘spelled exactly the same (apart from orthographic or grammatical corrections)’, as with homonyms, and that are ‘associated with the same type’, as with homotypic synonyms. Moreover, the question arises as to whether ‘different names’ here includes names that are ‘spelled exactly the same (apart from orthographic or grammatical corrections)’. These issues are important because they affect our ability to denote relationships between names using these terms, particularly if these relationships affect issues such as the legitimacy and illegitimacy of names [1].

The glossary of the Madrid revision of the *International Code of Nomenclature of Algae, Fungi and Plants* (https://www.iaptglobal.org/_functions/code/madrid; accessed 18 November 2025) defines the term ‘isonym’ as follows:

‘*isonym*. The same name based on the same type, published independently at different times by the same or different authors.’

In 2019, Tindall [14] proposed a similar definition of ‘isonyms’ to be included into the ICNP:

‘The same name, based on the same nomenclatural type, that has been validly published independently at different times, possibly by different authors are isonyms [sic]’.

Although this proposal was not ratified by the ICSP [5], it provides valuable insights. As the term ‘same name’ is not defined in the ICNP, considerations of sameness, or identity, arise. The Oxford Learner’s Dictionary provides two definitions of the English adjective ‘same’ (https://www.oxfordlearnersdictionaries.com/definition/english/same_1?q=same; accessed 18 November 2025), a strict and a broad one. Sameness, or identity, in the strict sense is the ‘relation everything has to itself and to nothing else’ (https://plato.stanford.edu/entries/identity; accessed 18 November 2025). For instance, strict sameness may refer to two mentions of the same individual, while sameness in the broad sense may refer to someone being an identical twin of another person.

None of the above definitions of isonyms can derive the sameness (of names) they refer to from the names having the same type, as this would make the definitions pleonastic. Otherwise, they would imply that names could be the same (because they look the same), even if they had different types. Tindall’s proposed phrasing could not have referred to sameness (of names) in the strict sense of the word, so a clearer definition would be:

‘Names for taxa in the same category, spelled exactly the same (apart from orthographic or grammatical corrections, as detailed in Section 9) and based on the same nomenclatural type, that have been validly published independently at different times, possibly by different authors, are *isonyms*’.

Although Tindall [14] did not explicitly state that later isonyms are illegitimate, he proposed that only ‘the earliest validly published isonym is to be taken into consideration when considering priority of publication and selection of the authorship and date of valid publication’. Comparing this with Principles 6–8, Rule 23a and particularly Rule 23b of the ICNP [2,3, 5] suggests that Tindall would indeed consider later validly published isonyms to be illegitimate. In fact, the proposed definition of ‘isonym’ would have rendered later validly published isonyms illegitimate under Rule 51b(1) of the ICNP [2,3, 5].

Therefore, it would be desirable to have a term to denote these kinds of illegitimate names, since the term ‘isonym’ is not included in the ICNP [2,3, 5]. According to the above definitions, isonyms cannot be categorised as homonyms or heterotypic synonyms. Are they homotypic synonyms? This depends on how ‘more than one name’ is interpreted in the definition of the term. For example, according to the broad definition of sameness mentioned earlier, *Budvicia aquatica* Bouvet *et al*. 1985 [15] and its later ‘isonym’, *Budvicia aquatica* Aldová *et al*. 1985 [16,17], are considered to be the same name. However, in the strict sense of sameness, *Budvicia aquatica* Bouvet *et al*. 1985 and *Budvicia aquatica* Aldová *et al*. 1985 are ‘more than one name’. This would allow *Budvicia aquatica* Aldová *et al*. 1985 to be considered a later homotypic synonym of *Budvicia aquatica* Bouvet *et al*. 1985. While this designation would not reveal the identical spelling of the two names, at least there would be a term to denote the relationship between them in the absence of a definition of ‘isonym’ in the ICNP [2,3, 5].

According to their position in the definitions of the two kinds of synonym under the ICNP, the phrases ‘more than one name’ and ‘different names’ have the same meaning. Assuming that ‘different names’ as well as ‘more than one name’ refer to sameness in the strict sense also allows for heterotypic synonyms to be ‘spelled exactly the same (apart from orthographic or grammatical corrections, as detailed in Section 9’. This, in turn, enables a situation such as that presented in the current Request for an Opinion [4] to be denoted, in which two names are both homonyms and heterotypic synonyms. The Judicial Commission concludes that the definitions for homonyms and synonyms provided in the ICNP [2,3, 5] suffice to cover the relationships between names that are of interest in this context, when the relevant kind of sameness or otherness of names is taken into account. As implied by Rule 28a, Opinion 128 [18] and the new Note 3 to Rule 56a [2,3], non-strict sameness is relevant to the rejection of names. The rejection of a name also places all of its homonyms (and isonyms, for that matter) on the list of rejected names. In contrast, the above definitions of homonyms and synonyms appear to refer to strict sameness of names [2,3, 5].

### The illegitimacy of the considered names

The Judicial Commission agrees with the Request for an Opinion [4] that *Methylacidiphilum* Awala *et al*. 2023 [12,13] is illegitimate because it is a later homonym of *Methylacidiphilum* Ratnadevi *et al*. 2023 [10,11]. This is stipulated in Rule 51b(5) of the ICNP [2,3, 5]. According to Rules 51a and 51b(2), the illegitimacy of the genus name also renders the name of its type and sole species, *Methylacidiphilum caldifontis* Awala *et al*. 2023, illegitimate, but not the epithet *caldifontis* (Rule 32b).

Another question is whether *Methylacidiphilaceae* Awala *et al*. 2023 and *Methylacidiphilales* Awala *et al*. 2023 are also illegitimate, as stated in the Request for an Opinion [4]. Notably, *Methylacidiphilum* Awala *et al*. 2023 is the type genus of the family and the order. An illegitimate name may not be used (Rule 51a); therefore, if it cannot be used in general, it cannot be used as the name of a type genus either. Moreover, the 2025 revision of the ICNP [2,3] explicitly lists this as a reason ‘for which a name may be illegitimate’ in Rule 51b(6): ‘If the nomenclatural type of the taxon to which the name was applied is a taxon with an illegitimate name itself (see Rules 20a, 21a and 51a)’. This would confirm that *Methylacidiphilaceae* Awala *et al*. 2023 and *Methylacidiphilales* Awala *et al*. 2023 are illegitimate names.

However, the wording of Rule 21a must be considered. According to this rule, the ‘nomenclatural type (see Rule 15) of a taxon above genus, up to and including order, is the included genus with a validly published and legitimate name on which the name of the relevant taxon is based’. Since *Methylacidiphilum* Awala *et al*. 2023 [12,13] and *Methylacidiphilum* Ratnadevi *et al*. 2023 [10,11] are homonyms and considered heterotypic synonyms [4], one might therefore conclude that *Methylacidiphilum* Ratnadevi *et al*. 2023 could be this ‘included genus with a validly published and legitimate name on which the name of the relevant taxon is based’ in case of the family and the order. If so, their names would not be illegitimate, meaning no action would be required concerning them. However, as will be shown, this would be a misinterpretation of Rule 21a.

Firstly, Rule 15 of the ICNP [2,3, 5] indicates that the ‘nomenclatural type, referred to in this Code as “type”, is that element of the taxon with which the name is permanently associated, whether as a correct name or as a synonym’. Therefore, a nomenclatural type must be permanently associated with the name for which it serves as the type. We can consider permanent association to be a transitive relation. Therefore, if A is permanently associated with B and B with C, then A is also permanently associated with C. However, *Methylacidiphilum* Ratnadevi *et al*. 2023 and *Methylacidiphilum* Awala *et al*. 2023 are only heterotypic synonyms and are therefore not permanently associated with each other for the purposes of the ICNP [2,3, 5].

As stipulated in its name and in General Consideration 4 and in Principle 1 (4), the ICNP does not rule on taxonomy. The definition of nomenclature, on which it actually rules, is given in General Considerations 4 and 7. Principle 8 Note 2 provides further relevant definitions. The assumption of heterotypic synonymy between *Methylacidiphilum* Ratnadevi *et al*. 2023 [10,11] and *Methylacidiphilum* Awala *et al*. 2023 [12,13] depends on taxonomic views regarding the circumscription of these genera. If they were considered to have a narrower circumscription, they would not be considered synonyms. *Methylacidiphilum* Awala *et al*. 2023 is permanently associated with *Methylacidiphilaceae* Awala *et al*. 2023 and *Methylacidiphilales* Awala *et al*. 2023, but *Methylacidiphilum* Ratnadevi *et al*. 2023 [10,11] is not. Much like the ICNP, the Judicial Commission does not rule on taxonomy and therefore cannot assume that assumptions on heterotypic synonyms automatically hold. A relationship that can change with taxonomic opinion at any time does not provide a permanent association.

In summary, *Methylacidiphilum* Ratnadevi *et al*. 2023 is currently not permanently associated with *Methylacidiphilaceae* Awala *et al*. 2023 or *Methylacidiphilales* Awala *et al*. 2023 and therefore cannot serve as their nomenclatural type. Under the ICNP, there is no way to be sure that *Methylacidiphilum* Ratnadevi *et al*. 2023 is the validly published and legitimate name of the genus *Methylacidiphilum* Awala *et al*. 2023.

Secondly, the crucial verbs ‘to include’ and ‘to base’, which are used in the first sentence of Rule 21a [2,3, 5] refer to the actions of the authors of a taxon name when proposing the name. They do not refer to subsequent actions, such as treating the type genus as a heterotypic synonym of another genus. While this may not be obvious from the phrasing of the sentence, it is a common theme in the ICNP.

For example, Rule 20c [2,3, 5] regulates the inference of the type species of a monotypic genus. This inference is based on an action of the authors (i.e. placing exactly one species in that genus), which is decisive: ‘If the genus, when its name is validly published, included only one species, then that species is the type species irrespective of whether it is designated as the type’. The inclusion of that sole species constitutes an action by the authors at the time of proposing the genus name. This decision of the authors is to be followed.

Rule 22 [2,3, 5] also allows for not explicitly designating the type if the higher taxon is monotypic: ‘If there is only one genus, this becomes the type. If there are two or more genera, the type shall be designated by the author(s) at the time of the proposal of the phylum name… If only one genus name is validly published, this becomes the type. If two or more genera have validly published names, the type shall be designated by the author(s) at the time of the proposal of the name’. The inclusion of this sole genus is done by the authors at the time of proposing the name of the higher taxon. Again, the decision of the authors is to be followed.

The inclusion of genera in families, suborders or orders is an action conducted by the authors of the proposed taxon name. They have to provide a description (‘protologue’) or refer to a previously published one (Rule 27), which must list at least one genus [2,3, 5]. Designating the type genus is an action conducted by the authors of the name of the proposed taxon. This can only be omitted if the type genus can be inferred because the family, suborder or order is monotypic (Rule 20c).

*Methylacidiphilum* Awala *et al*. 2023 is illegitimate, but this does not make *Methylacidiphilum* Ratnadevi *et al*. 2023 ‘the included genus with a validly published and legitimate name on which the name of the relevant taxon is based’. Since *Methylacidiphilum* Ratnadevi *et al*. 2023 is a homonym of *Methylacidiphilum* Awala *et al*. 2023, *Methylacidiphilaceae* Awala *et al*. 2023 and *Methylacidiphilales* Awala *et al*. 2023 could, in theory, have been based on the name of the former genus. However, Awala *et al*. based the names *Methylacidiphilaceae* Awala *et al*. 2023 and *Methylacidiphilales* Awala *et al*. 2023 on the name *Methylacidiphilum* Awala *et al*. 2023, not on the name *Methylacidiphilum* Ratnadevi *et al*. 2023. Nor did they include *Methylacidiphilum* Ratnadevi *et al*. 2023 into the family or the order. This is because they were not aware of *Methylacidiphilum* Ratnadevi *et al*. 2023 at the time of writing.

Additional sentences in the 2025 revision [2,3] support this conclusion, although they merely summarise existing Rules of the ICNP [5]. Rule 16 now states that the ‘type of a taxon must be designated by the author(s) at the time the name of the taxon is published in the IJSEM [see Rules 15, 18a, b, f, 20a-c, 21a, 22 and 27(3)], unless the type of the taxon can be inferred according to Rules 20c, 21a, 21b or 22’. This means that authors usually have to designate the type; otherwise, they may contravene this rule. Authors can only omit designating the type without contravening Rule 16 if it can be inferred. It is normal practice for authors to designate the type. Exceptions are implemented only to allow the omission of the type designation under defined circumstances. If the authors have designated a type, their decision must be followed. This is also stipulated in Rule 16a Note 3: ‘A type designated by the authors, or designated by the authors and replaced according to Rule 22 paragraph 2, or not designated by the authors and inferred as indicated above must be accepted, unless it is replaced in accordance with Rules 18c, 18f, 18g or 37a(2)’.

Notably, Rule 21a [2,3, 5] also does not provide for the ad hoc replacement of a type genus with an illegitimate name by a type genus with a legitimate name. This is made clearer by comparing Rule 21a with a clause that provides an automated replacement mechanism, such as Rule 22 paragraph 2. Apart from Rule 22 paragraph 2, there is no mechanism in the code by which a nomenclatural type gets automatically replaced.

In summary, Awala *et al*. [12,13] have based neither *Methylacidiphilaceae* Awala *et al*. 2023 nor *Methylacidiphilales* Awala *et al*. 2023 on *Methylacidiphilum* Ratnadevi *et al*. 2023, nor did they include this genus in the family or order. Consequently, *Methylacidiphilum* Ratnadevi *et al*. 2023 is not currently the nomenclatural type of the family or order.

Therefore, Rule 21a is relevant to the present case because if the designated or inferred type genus has an illegitimate name, then the name of the higher taxon for which that genus is the type also contravenes Rule 21a and is illegitimate. This conclusion is also drawn in the underlying Request for an Opinion [4]. The actual implication of Rule 21a is that if *Methylacidiphilum* Awala *et al*. 2023 is illegitimate, then so are *Methylacidiphilaceae* Awala *et al*. 2023 and *Methylacidiphilales* Awala *et al*. 2023.

### Resolving the illegitimacy of the considered names

As detailed above, the names *Methylacidiphilum caldifontis* Awala *et al*. 2023, *Methylacidiphilum* Awala *et al*. 2023, *Methylacidiphilaceae* Awala *et al*. 2023 and *Methylacidiphilales* Awala *et al*. 2023 [12,13] are illegitimate. The Judicial Commission also agrees with the Request for an Opinion [4] that the names *Methylacidiphilaceae* Awala *et al*. 2023 and *Methylacidiphilales* Awala *et al*. 2023 cannot be replaced by taxonomic action based on *Methylacidiphilum* Ratnadevi *et al*. 2023 [10,11] as the type genus, as this would create later homonyms that would be illegitimate according to Rule 51b(5). The question therefore arises as to whether the Judicial Commission should take action.

The 2025 revision of the Code [2,3] makes it clearer than before that conservation of names by the Judicial Commission is not only a means of conserving one name over another. The new Note 4 to Rule 56b states that ‘information on conserving a name for the purpose of changing its nomenclatural type’ is given in Rule 37a(2). This rule in turn stipulates that the retention ‘of a name in a sense that excludes the type can only be effected by conservation and only by the Judicial Commission (see also Rule 23a). At the time of conservation, the new type is established by the Judicial Commission’. The central Rule 23a has also been expanded to stipulate that a ‘conserved name (*nomen conservandum*) is a name that must be used instead of all earlier synonyms and homonyms… Alternatively, a name can be conserved to change its nomenclatural type [see Rule 37a(2)]’.

This means that the removal of *Methylacidiphilum* Awala *et al*. 2023, a genus with an illegitimate name, as type of *Methylacidiphilaceae* Awala *et al*. 2023 and *Methylacidiphilales* Awala *et al*. 2023 [12,13] would be possible, if another suitable nomenclatural type is immediately assigned. The Judicial Commission therefore has three paths of action.

Firstly, doing nothing. This means that a name at the family level for the genus *Methylacidiphilum* Ratnadevi *et al*. 2023 [10,11] remains unavailable, except in taxonomic treatments that assign the genus to a different family. Unless taxonomists assign the genus to a family with an already validly published and legitimate name, it will be necessary to wait for a proposal for a family containing *Methylacidiphilum* Ratnadevi *et al*. 2023, based on a different genus. The same applies to the order level. Moreover, *Methylacidiphilum caldifontis* Awala *et al*. 2023 [12,13] would also remain unavailable, necessitating a replacement name if the species is considered a separate species.

Secondly, conserve *Methylacidiphilum* Awala *et al*. 2023 [12,13] over *Methylacidiphilum* Ratnadevi *et al*. 2023 [10,11] as suggested in the Request for an Opinion [4]. This would render *Methylacidiphilum* Ratnadevi *et al*. 2023 and *Methylacidiphilum kamchatkense* Ratnadevi *et al*. 2023 unavailable, necessitating a replacement name for *Methylacidiphilum kamchatkense* Ratnadevi *et al*. 2023 if considered a separate species. However, it would make *Methylacidiphilum caldifontis* Awala *et al*. 2023 available.

Thirdly, conserve *Methylacidiphilaceae* Awala *et al*. 2023 and *Methylacidiphilales* Awala *et al*. 2023 [12,13] with *Methylacidiphilum* Ratnadevi *et al*. 2023 [10,11] as nomenclatural type. This would also make the names at the rank of family and order legitimate. *Methylacidiphilum caldifontis* Awala *et al*. 2023 would remain unavailable in this scenario, again necessitating a replacement name if this species is considered a separate species. However, *Methylacidiphilum* Ratnadevi *et al*. 2023 and *Methylacidiphilum kamchatkense* Ratnadevi *et al*. 2023 would remain legitimate names.

In the second scenario, the replacement name to be proposed would be *Methylacidiphilum kamchatkense* (Ratnadevi *et al*. 2023) comb. nov., placing that species in *Methylacidiphilum* Awala *et al*. 2023. In the third scenario, the replacement name to be proposed would be *Methylacidiphilum caldifontis* (Awala *et al*. 2023) comb. nov., placing that species in *Methylacidiphilum* Ratnadevi *et al*. 2023. Notably, these names would be illegitimate according to Rule 51b(5) if they were later homonyms of their respective basonym. However, the newly introduced definition of homonyms, as discussed above, requires homonyms to be based on different types [2,3], so this problem would not arise. It should be noted, however, that any taxonomist could propose replacement names, and the Judicial Commission does not decide on taxonomy.

The Judicial Commission therefore first decided whether or not to take action. Eleven commissioners voted in favour of doing so, one voted against it and none abstained. A second ballot was then held to decide which of the following solutions should be adopted if action was to be taken: (1) *Methylacidiphilum* Awala *et al*. 2023 [12,13] should be conserved over *Methylacidiphilum* Ratnadevi *et al*. 2023 [10,11] or (2) *Methylacidiphilaceae* Awala *et al*. 2023 and *Methylacidiphilales* Awala *et al*. 2023 [12,13] should be conserved with *Methylacidiphilum* Ratnadevi *et al*. 2023 [10,11] as the nomenclatural type. No commissioners voted in favour of the first option, ten in favour of the second, one commissioner abstained and one commissioner did not participate in this ballot. A third ballot was devoted to the question of whether a replacement species name should be proposed directly in the Judicial Opinion. Eleven commissioners voted in favour of directly proposing a replacement name, none voted against it, none abstained and one commissioner did not participate in this ballot.

## Taxonomic consequences

### Description of *Methylacidiphilum caldifontis* comb. nov.

cal.di.fon’tis. L. masc. adj. *caldus*, hot; L. masc. n. *fons*, a spring; N.L. gen. masc. n. *caldifontis*, of a hot spring.

Illegitimate basonym: *Methylacidiphilum caldifontis* Awala *et al*. 2023 [12,13].

The description of the species is as given for *Methylacidiphilum caldifontis* Awala *et al*. 2023 [12,13]. The type strain is IT6^T^ (=KCTC 92103^T^=JCM 39288^T^), which was isolated from a subaqueous mud sample taken from Pisciarelli hot spring (40° 49′ 45.1′′ N 14° 08′ 49.7′′E) in Pozzuoli, Italy.

## Opinion 134

### The cases presented

The Request for an Opinion by Li [6] covers two topics: one concerning the synonymy between *Yokenella regensburgei* Kosako *et al*. 1985 [17,19] and *Koserella trabulsii* Hickman-Brenner *et al*. 1985 [17,20], and the other concerning the synonymy between *Providencia alcalifaciens* (de Salles Gomes 1944) Ewing 1962 (Approved Lists 1980) [21,22] and *Proteus inconstans* (Ornstein 1920) Shaw and Clarke 1955 (Approved Lists 1980) [22,23]. Apart from raising issues of priority, the two cases have only some aspects in common and will be considered separately below.

### *Yokenella regensburgei* vs. *Koserella trabulsii*

The key points from Li’s Request for an Opinion [6] in this context are as follows:

- *Yokenella regensburgei* Kosako *et al*. 1985 [17,19] and *Koserella trabulsii* Hickman-Brenner *et al*. 1985 [17,20] were validly published by inclusion in the same Validation List.
- This Validation List [17] was published before sequence numbers, reflecting the date of receipt of each validation request, were required to be included in Validation Lists, in order to determine priority when names compete for it [24].
- The study that first considered the two species names to be heterotypic synonyms [25] did not select one of the names as the correct one over the other.
- A later study [26] showed that the validation request for *Yokenella regensburgei* Kosako *et al*. 1985 [17,19] had been submitted earlier than that for *Koserella trabulsii* Hickman-Brenner *et al*. 1985 [17,20].
- The Judicial Commission [27] argued that if sequence numbers were missing but ‘it can be demonstrated that the request for validation of one of these names was submitted before the other, the one of earliest submission would have been entitled to the lower sequence number and will have priority upon simple publication of the proof’.
- Until the 2008 revision of the ICNP [28], the Code did not specify how priority should be determined when Validation Lists did not contain sequence numbers. This was added to Rule 24b in the 2022 revision [5] following a proposal by Tindall [29]. This retroactively states that ‘the author who first unites the taxa has the right to choose one of them, and this choice must be followed’.
- However, this leaves the question of the priority between the names open, as the study that first united the two species did not make that choice.
- Following the publications in 1991 [26,27], the name *Yokenella regensburgei* Kosako *et al*. 1985 was more frequently used than *Koserella trabulsii* Hickman-Brenner *et al*. 1985.
- Contemporary methods for species delineation may well conclude that the two names refer to the same species [6].
- The Judicial Commission’s guidelines [1] indicate that this is not a case for rejecting one of these names, but rather a case for conserving one name over another.
- In order to stabilize the nomenclature of these bacteria, the genus name and epithet of *Yokenella regensburgei* Kosako *et al*. 1985 would be better conserved over those of *Koserella trabulsii* Hickman-Brenner *et al*. 1985.

The Judicial Commission agrees with this assessment. As the name *Yokenella regensburgei* Kosako *et al*. 1985 is already preferred to *Koserella trabulsii* Hickman-Brenner *et al*. 1985, there is no particular urgency to resolve this issue. However, the priority of the former over the latter could be called into question in the future. Both names are associated with a risk group of two in several countries but *Yokenella regensburgei* Kosako *et al*. 1985 is the more frequently used name [30]. Bartlett *et al*. [31] list *Yokenella regensburgei* Kosako *et al*. 1985 as an established human pathogen but not *Koserella trabulsii* Hickman-Brenner *et al*. 1985. The stability of the names of taxa of possible or established medical relevance is of particular interest [32]. The conservation of names or epithets is intended to resolve cases of unwanted priority and does not presuppose that any of the taxon names involved have intrinsic problems [1]. Conversely, the Judicial Commission is not aware of any disadvantages to conserving the more frequently used name, particularly in light of the 1991 assessment [26,27]. The 2025 revision of the Code [2,3] did not change Rule 24a with regard to the aspects relevant to the priority between these names.

However, the 2025 revision [2,3] added examples to Rules 23a and 42, which further clarify that the priority of epithets must be considered separately from that of the generic name. Notes were also added to Rules 56a and 56b to clarify that the rejection and conservation mechanisms work slightly differently for epithets and generic names. Conserving *Yokenella* Kosako *et al*. 1985 over *Koserella* Hickman-Brenner *et al*. 1985 would not affect the priority of the epithets; therefore, one might still argue that *Yokenella trabulsii* [Hickman-Brenner et al. 1985] should be proposed. For this reason, if conservation is to be conducted, it should be done separately for the generic name and the epithet.

The Judicial Commission therefore grants the request [6] to conserve the name *Yokenella* Kosako *et al*. 1985 over *Koserella* Hickman-Brenner *et al*. 1985 as well as the epithet in *Yokenella regensburgei* Kosako *et al*. 1985 over the epithet in *Koserella trabulsii* Hickman-Brenner *et al*. 1985. Twelve commissioners voted in favour of this conclusion, none voted against it and none abstained. The Judicial Commission did not consider the part of the request [6] that called for conservation ‘with a particular circumscription’, as this constitutes a different kind of conservation. The 2025 revision of the Code [2,3] provides further clarification on this issue in the Notes to Rules 23a and 56b.

### *Providencia alcalifaciens* vs. *Proteus inconstans*

In its second part, the Request for an Opinion by Li [6] presents the following argumentation:

- The species *Providencia alcalifaciens* (de Salles Gomes 1944) Ewing 1962 (Approved Lists 1980) [21,22] is the nomenclatural type of *Providencia* Ewing 1962 (Approved Lists 1980) [21,22].
- The names *Providencia alcalifaciens* (de Salles Gomes 1944) Ewing 1962 (Approved Lists 1980) and *Proteus inconstans* (Ornstein 1920) Shaw and Clarke 1955 (Approved Lists 1980) [22,23] were included in the Approved Lists with the same nomenclatural type (ATCC 9886).
- This makes the name *Providencia alcalifaciens* (de Salles Gomes 1944) Ewing 1962 (Approved Lists 1980) a later homotypic synonym of *Proteus inconstans* (Ornstein 1920) Shaw and Clarke 1955 (Approved Lists 1980), and the epithet *inconstans* has priority over the epithet *alcalifaciens*.
- According to Rule 51b(2) of the ICNP, this renders *Providencia alcalifaciens* (de Salles Gomes 1944) Ewing 1962 (Approved Lists 1980) illegitimate.
- According to Rule 20a of the ICNP, this renders *Providencia* Ewing 1962 (Approved Lists 1980) illegitimate, and thereby also renders all other validly published names of species placed in *Providencia* illegitimate.
- Furthermore, the name *Providencia alcalifaciens* (de Salles Gomes 1944) Ewing 1962 (Approved Lists 1980) is more widely used than *Proteus inconstans* (Ornstein 1920) Shaw and Clarke 1955 (Approved Lists 1980).
- In order to stabilize the nomenclature of these bacteria by rendering the genus name *Providencia* Ewing 1962 (Approved Lists 1980) legitimate, the epithet of *Providencia alcalifaciens* (de Salles Gomes 1944) Ewing 1962 (Approved Lists 1980) would be better conserved over the one of *Proteus inconstans* (Ornstein 1920) Shaw and Clarke 1955 (Approved Lists 1980).

While the Judicial Commission partially agrees with this argumentation, there are some aspects that require a more in-depth consideration. According to Rule 24a Note 2 [2,3, 5], the presence of homotypic synonyms in the Approved Lists is not unusual in itself. As mentioned above, the priority of epithets must be considered separately from that of the generic name. In other words, there can be no doubt that the epithet *inconstans* has priority over the epithet *alcalifaciens*. However, regarding the illegitimacy of the name *Providencia alcalifaciens* (de Salles Gomes 1944) Ewing 1962 (Approved Lists 1980), it must be taken into account that Rule 51b(2) refers to a situation in which ‘the author(s) did not adopt for a … combination the earliest legitimate … specific epithet … available for the taxon’. This does not appear to apply to a case in which the synonymy was created when the Approved Lists [22] were compiled rather than by the original authors.

Rule 24a Note 5 of the 2025 revision [2,3] now explicitly clarifies the status of basonyms in the Approved Lists. The basonym of *Providencia alcalifaciens* (de Salles Gomes 1944) Ewing 1962 (Approved Lists 1980) is ‘*Eberthella alcalifaciens*’ de Salles Gomes 1944 [33]. The author did not assign a type strain. However, Ewing [21] referenced ATCC 9886 as representing the culture examined by de Salles Gomes 1944 [33], probably explaining why it was chosen as the nomenclatural type by the Approved Lists [22]. The basonym of *Proteus inconstans* (Ornstein 1920) Shaw and Clarke 1955 (Approved Lists 1980) is ‘*Bacillus inconstans*’ Ornstein 1920 [34]. Apparently, de Salles Gomes 1944 [33] was not aware of the work of Ornstein [34]. Ewing [21] was aware of that work but explicitly dismissed the use of the epithet *inconstans*. Shaw and Clarke [23], who opined that the ‘Providence group’ of bacteria should be affiliated with the genus *Proteus* [22,35], noted that ‘NCTC 2481, *Bacillus inconstans* Ornstein, in our routine examination was found to have the biochemical characters of the Providence group. Dr W. H. Ewing confirmed this and has also shown that serologically the strain belongs to Providence 0 group 9’. However, Ewing [21] stated that ‘the strain (NCTC 2481) was not an original Ornstein culture, but one identified as *B. inconstans* Ornstein by other investigators’.

The Approved Lists [22] may also have assigned ATCC 9886 as the type strain of *Proteus inconstans* (Ornstein 1920) Shaw and Clarke 1955 (Approved Lists 1980), since this species was known to represent the ‘Providence group’ and the editors intended to reflect the differing views on its genus affiliation in accordance with Rule 24a Note 2 [2,3, 5]. This affiliation could be either to a genus of its own, *Providencia* Ewing 1962 (Approved Lists 1980) [21,22], or to *Proteus* [22,35]. Regardless of the historical details, these considerations cast doubt on the conclusion that *Providencia alcalifaciens* (de Salles Gomes 1944) Ewing 1962 (Approved Lists 1980) is illegitimate according to Rule 51b(2) of the ICNP [2,3, 5]. Assuming *Providencia* Ewing 1962 (Approved Lists 1980) is legitimate, priority of the epithets would warrant the creation of a new combination, *Providencia inconstans* (Ornstein 1920). While this would be considered the correct name for what is currently known as *Providencia alcalifaciens* (de Salles Gomes 1944) Ewing 1962 (Approved Lists 1980), it would not affect the status of the latter as the type species of *Providencia* Ewing 1962 (Approved Lists 1980) according to Rule 15 [2,3, 5].

If so, are there any other reasons for conserving the epithet *alcalifaciens* over *inconstans* in these names? As mentioned above, the conservation of names or epithets is intended to resolve cases of unwanted priority [1]. This usually involves conserving the better known (more frequently used) name or epithet over the less well known (rarely used) name or epithet [36,37]. *Providencia alcalifaciens* (de Salles Gomes 1944) Ewing 1962 (Approved Lists 1980) and *Proteus inconstans* (Ornstein 1920) Shaw and Clarke 1955 (Approved Lists 1980) are both associated with a risk group of two in several countries but *Providencia alcalifaciens* (de Salles Gomes 1944) Ewing 1962 (Approved Lists 1980) is the more frequently used name [30]. Bartlett *et al*. [31] list *Providencia alcalifaciens* (de Salles Gomes 1944) Ewing 1962 (Approved Lists 1980) as an established human pathogen but not *Proteus inconstans* (Ornstein 1920) Shaw and Clarke 1955 (Approved Lists 1980). As mentioned above, the stability of the names of taxa of possible or established medical relevance is of particular concern [32].

The Judicial Commission therefore grants the request [6] to conserve the epithet in *Providencia alcalifaciens* (de Salles Gomes 1944) Ewing 1962 (Approved Lists 1980) over the epithet in *Proteus inconstans* (Ornstein 1920) Shaw and Clarke 1955 (Approved Lists 1980). Twelve commissioners voted in favour of this conclusion, none voted against it and none abstained.

## Abbreviations

ICNP: International Code of Nomenclature of Prokaryotes
ICSP: International Committee on Systematics of Prokaryotes
